# MiR-223 regulates the differentiation of immature neurons

**DOI:** 10.1186/2052-8426-2-18

**Published:** 2014-06-17

**Authors:** Maged M Harraz, Jin-Chong Xu, Noah Guiberson, Ted M Dawson, Valina L Dawson

**Affiliations:** Solomon H. Snyder Department of Neuroscience, Johns Hopkins University School of Medicine, Baltimore, MD 21205 USA; Department of Histology and Genetics, Suez Canal University School of Medicine, Ismailia, Egypt; Neuroregeneration and Stem Cell Programs, Institute for Cell Engineering, Johns Hopkins University School of Medicine, 733 North Broadway, BRB 731 21205 Baltimore, MD USA; Department of Neurology, Johns Hopkins University School of Medicine, Baltimore, MD 21205 USA; Department of Physiology, Johns Hopkins University School of Medicine, Baltimore, MD 21205 USA; Department of Pharmacology and Molecular Sciences, Johns Hopkins University School of Medicine, Baltimore, MD 21205 USA

**Keywords:** Neural stem cells, microRNA, Dendrite development

## Abstract

**Background:**

Small non-coding microRNA RNA molecules can regulate stem cell function. The role of microRNAs in neural stem/progenitor cells (NS/PCs) differentiation is not entirely clear.

**Methods:**

MiRNA profiling, loss and gain of function studies coupled with dendritic tree development morphometric analysis and calcium influx imaging were utilized to investigate the role of micoRNA-223 in differentiating NS/PCs.

**Results:**

MiRNA profiling in human NS/PCs before and after differentiation in vitro reveals modulation of miRNAs following differentiation of NS/PCs. MiR-223, a microRNA well characterized as a hematopoietic-specific miRNA was identified. Cell-autonomous inhibition of miR-223 in the adult mouse dentate gyrus NS/PCs led to a significant increase in immature neurons soma size, dendritic tree total length, branch number per neuron and complexity, while neuronal migration in the dentate gyrus remained unaffected. Overexpression of miR-223 decreased dendritic tree total length, branch number and complexity in neurons differentiated from human embryonic stem cells (hESCs). Inhibition of miR-223 enhanced N-methyl-D-aspartate (NMDA) induced calcium influx in human neurons differentiated from NS/PCs.

**Conclusions:**

Taken together, these findings indicate that miR-223 regulates the differentiation of neurons derived from NS/PCs.

**Electronic supplementary material:**

The online version of this article (doi:10.1186/2052-8426-2-18) contains supplementary material, which is available to authorized users.

## Background

The neuronal processes of growth and branching during neural circuitry formation are regulated by multiple cell and non-cell autonomous mechanisms. Neurogenesis occurs during embryogenesis and is retained in certain areas of the adult brain including the dentate gyrus of the hippocampus and the subventricular zone [1]. Understanding neurogenesis opens exciting possibilities for cell transplantation therapy in neuro-traumatic, neurodegenerative and cerebrovascular diseases [2]. The long-term survival and integration of NS/PCs derived neurons into the existing neuronal circuitry is a pre-requisite for the success of stem cell therapy [3].

Regulation of protein translation plays an important role in the development of neural circuits; neural differentiation, dendrite development, synaptic plasticity and neural excitability [4]. MicroRNAs (miRNAs) are conserved small untranslated RNA molecules, which regulate protein synthesis. MiRNAs serve as a sequence specific guide for the RNA-induced silencing complex (RISC) to recognize its target mRNAs resulting in translation inhibition and/or mRNA degradation [5]. MicroRNAs play major roles in stem cell proliferation [6] and differentiation [7]. Multiple studies demonstrate the importance of miRNAs in the regulation of specific NS/PCs functions. For example miR-124 increases neuronal differentiation of adult NS/PCs [8]. Members of the miR-200 family on the other hand regulate olfactory neurogenesis [9]. MiR-9, another neuronal specific miRNA specifies sensory organ precursors in *Drosophila* [10], and is also involved in regulation of NS/PCs proliferation [11]. MiR-132 and miR-125b regulate dendritic spine morphology and synaptic function [12], and miR-375 regulates dendrite density [13]. In addition, miR-132 and miR-137 regulate dendritic growth and morphogenesis [14–16]. However, the role of microRNAs and the full range of microRNAs involved in the regulation of dendritic tree development remain poorly characterized.

## Methods

### Ethics statement

All experimental protocols were approved by the Johns Hopkins Institutional Review Board (IRB). All the animal handling and procedures were conducted in accordance with the National Institutes of Health guidelines for use of experimental animals and the Johns Hopkins animal care and use guidelines and approved protocols. The protocols were approved by the Johns Hopkins Institutional Animal Care and Use Committee (IACUC).

### Human NS/PC culture

Human NS/PCs were obtained from Lonza Walkersville, Inc. Walkersville, MD and cultured according to the supplier instructions. Neural progenitor cells were cryopreserved as neurospheres isolated from fetal human brain cortex. Differentiation was induced by withdrawing EGF/FGF2 and plating the cells on laminin + BDNF in Lonza neural progenitor differentiation media for 10 days.

### Gene ontology analysis

Gene functional annotation clustering was performed using The Database for Annotation, Visualization and Integrated Discovery (DAVID) v6.7 using the total set of TargetScan predicted human targets for hsa-miR-223. The human genome was used as a genetic background. P-values represent a modified Fisher’s exact test (EASE score) [17].

### Taqman low density quantitative PCR array

Total RNA was isolated using the Qiagen miRNeasy kit following the manufacturer instructions. A total of 50 ng of total RNA were used for reverse transcription using the Megaplex™ human pool A primers (Applied Biosystems, ABI). MicroRNA profiling was done after amplification of the cDNA using the ABI Megaplex™ preamp human pool A primers by Applied Biosystem’s Taqman low density quantitative PCR array (TLDA). TLDA was performed and data analyzed following the manufacturer instructions. The global mean normalization method was used to normalize the miRNA microarray data as described previously [18]. Briefly, all Ct values above 33 were considered noise and excluded from further analysis. The average Ct value for each sample was subtracted from each Ct value for that sample. Fold change of differentiated (D) relative to undifferentiated (U) was calculated with the following equation (using the normalized Ct values): Fold change = 2^-[Ct.D­-(Ct.U)]. Data were clustered using Cluster 3.0 and heat map was generated using Java TreeView version 1.1.4r3.

### Methods for microRNA, lentivirus and retrovirus

Approximately 600 bp fragment including the mouse genomic miR-223 locus driven by the U6 promoter or a non-targeting miRNA control were inserted into a lentiviral vector. It is important to note that the human and mouse miR-223 sequences are 100% identical. Production of lentiviral and retroviral vectors was conducted as described previously [19]. Anti-miR-223 sponge: A tandem repeat of miR-223 binding site 5′ TAGAACTGACAgcaaGGGGTATTT 3′ or mismatched sponge sequence 5′ TAGAACCAGCAgcaaGGGTGCTTT 3′ were cloned 3′ to the EGFP reporter in the lentiviral and retroviral vectors. We have previously verified targeting of miR-223 by the anti-miR-223 sponge but not the mismatch sponge [19].

### Stereotaxic injection of retrovirus into the adult mouse dentate gyrus

Stereotaxic injections were performed as described previously [20]. Briefly, a total of 4 injections per animal using 0.5 microliters total volume in each were made at co-ordinates for the dentate gyrus: from the bregma Y −2.0 mm, X +/− 1.6 mm, Z 2.5 mm and Y −3.0 mm X +/− 2.6 mm Z 3.2 mm.

### Neuronal morphometric analysis

For the adult dentate gyrus experiments: 10 mice were injected with mismatch sponge retrovirus and 15 mice were injected with anti-miR-223 sponge retrovirus in at least 3 independent experiments. In each experiment the investigator who analyzed the data was blinded to the experimental groups. Two weeks post-injection, the mice were sacrificed and their brains were processed for confocal image analysis. Each dentate gyrus neuron dendritic tree was imaged in a 30–40 z-series stacks with one-micrometer interval. For analysis of dendritic tree development in both adult dentate gyrus experiments and human NS/PCs experiments, 3D reconstruction of entire dendritic processes of each neuron was made from the z-series stacks of confocal images. The 2D projection images were then traced using the NIH ImageJ program with NeuronJ plugin. Total dendritic length and branch number per neuron were analyzed as described previously [20]. Soma size was measured using the Zeiss LSM5 software. Sholl analysis was performed using the NIH ImageJ Sholl analysis plugin as described previously [20].

### Calcium mobilization assay

Live confocal microscope imaging was used to monitor the calcium indicator dye Fluo-4 intensity in response to NMDA stimulation in human NS/PCs derived neurons transduced with lentiviral vectors expressing either anti-miR-223 sponge or mismatched sponge control. Briefly, neurons cultured on glass coverslips were loaded with Fluo-4 dye in culture media for 30 minutes. Neurons were placed in a 37°C heated adaptor on the confocal microscope in HBSS + 2 mM calcium chloride + 10 μM glycine. A 10-minute time series images were taken every 10 seconds starting with 2 minutes to establish a baseline then 100 μM NMDA was added. Areas of increased fluorescence following NMDA stimulation were quantified using Zeiss LSM5 software.

### Human ESCs derived NS/PCs differentiation

Human cortical neurons were derived from the H1 embryonic stem cell (ESC) line using our recently described method [21] and validated by immunostaining. Human cortical neurons derived from H1 ESCs by targeted differentiation for 60 days were immunopositive for the neuronal marker MAP2 and synaptic marker synaptophysin. Neurons were immunopositive for cortical layer-specific markers (TBR1 (layers I, V and VI), BRN2 (layers II-IV), SATB2 (layers II-IV, V), and CTIP2 (layer V and VI)).

### Statistical analysis

For statistical significance comparisons the following tests were used: Two groups; student’s *t*-test. Cumulative frequency; Kolmogorov-Smirnov test. Sholl analysis; two-way ANOVA.

## Results and discussion

### A general upregulation of miRNAs including miR-223 following NS/PCs differentiation

**Figure 1:**
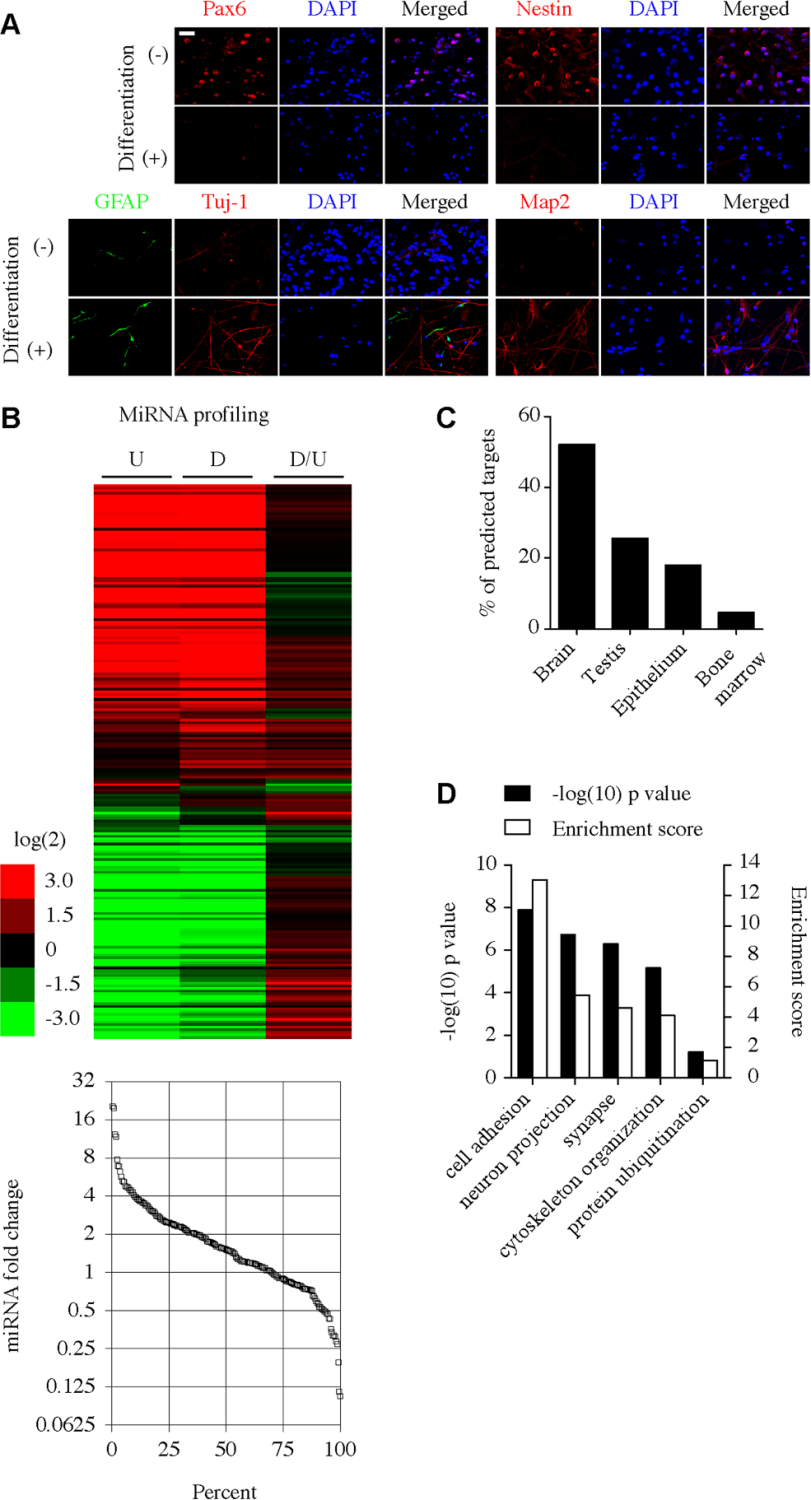
**General upregulation of miRNAs following NS/PCs differentiation. (A)** Immunocytochemistry for the undifferentiated and differentiated hNS/PCs. **(B)** Upper panel: Taqman low-density array (TLDA) analysis. Relative expression levels (heat-map) after global mean normalization are shown as log2 ratios for undifferentiated “U”, differentiated “D” and the fold change in differentiated relative to undifferentiated “D/U” hNS/PCs. TLDA was performed in 2 independent experiments. Lower panel: a plot demonstrating the fold change of individual miRNAs. Each square represents one miRNA. **(C)** Select tissue distribution comparison for human miR-223 predicted targets. **(D)** Gene ontology analysis for human brain-expressed miR-223 predicted targets. The left Y-axis represents the EASE score (Expression Analysis Systematic Explorer; a modified Fisher Exact *p*-Value) *p*-values for gene-enrichment annotation cluster terms. The right Y-axis represents the cluster enrichment scores.

Inducing the differentiation of NS/PCs in vitro led to upregulation of the neuronal markers Tuj-1 & MAP2, and downregulation of NS/PCs markers Nestin & Pax6 (Figure 1A). A quantitative PCR-based microarray was used to determine the expression profile of miRNAs in human NS/PCs before and after differentiation (Figure 1B). The global mean normalization method was used as a reference for fold change of miRNA levels. A general upregulation of microRNAs was observed following differentiation of normal human NS/PCs in vitro (Figure 1B). Out of 216 consistently detectable miRNAs, 108 were upregulated (>1.5 fold), 72 were unchanged (>0.75 < 1.5 fold) and 36 were downregulated (<0.75 fold) (Figure 1B). Multiple upregulated miRNAs are known to be associated with neuronal differentiation such as miR-124, and miR-200c. In addition, many upregulated miRNAs that are not well characterized to be associated with neural differentiation are identified, including miR-223. However, the role of miR-223 in NS/PCs differentiation is not known. On average miR-223 was upregulated 2.8-fold following human NS/PCs differentiation. To determine potential targets for miR-223 in human NS/PCs differentiation, the targetscan algorithm was used to generate a list of total predicted human targets irrespective of site conservation (Additional file 1: Dataset S1). Next this list was analyzed using the database for annotation, visualization and integrated discovery (DAVID) version 6.7. DAVID bioinformatics analysis revealed that the majority of human miR-223 predicted targets are expressed in the brain (Figure 1C and Additional file 1: Dataset S1), a finding that might suggest a functional role for miR-223 in the CNS. Further gene ontology analysis suggested that miR-223’s potential targets regulate important functions such as cell adhesion, neuron projection development, synapses, and cytoskeleton organization, but not cell migration or protein ubiquitination (Figure 1D and Additional file 1: Dataset S1). These results suggest that miR-223 is expressed during NS/PCs differentiation and that it could regulate immature neuron morphogenesis and function.

### MiR-223 regulates soma size and dendritic development but not migration of dentate gyrus adult born neurons

**Figure 2:**
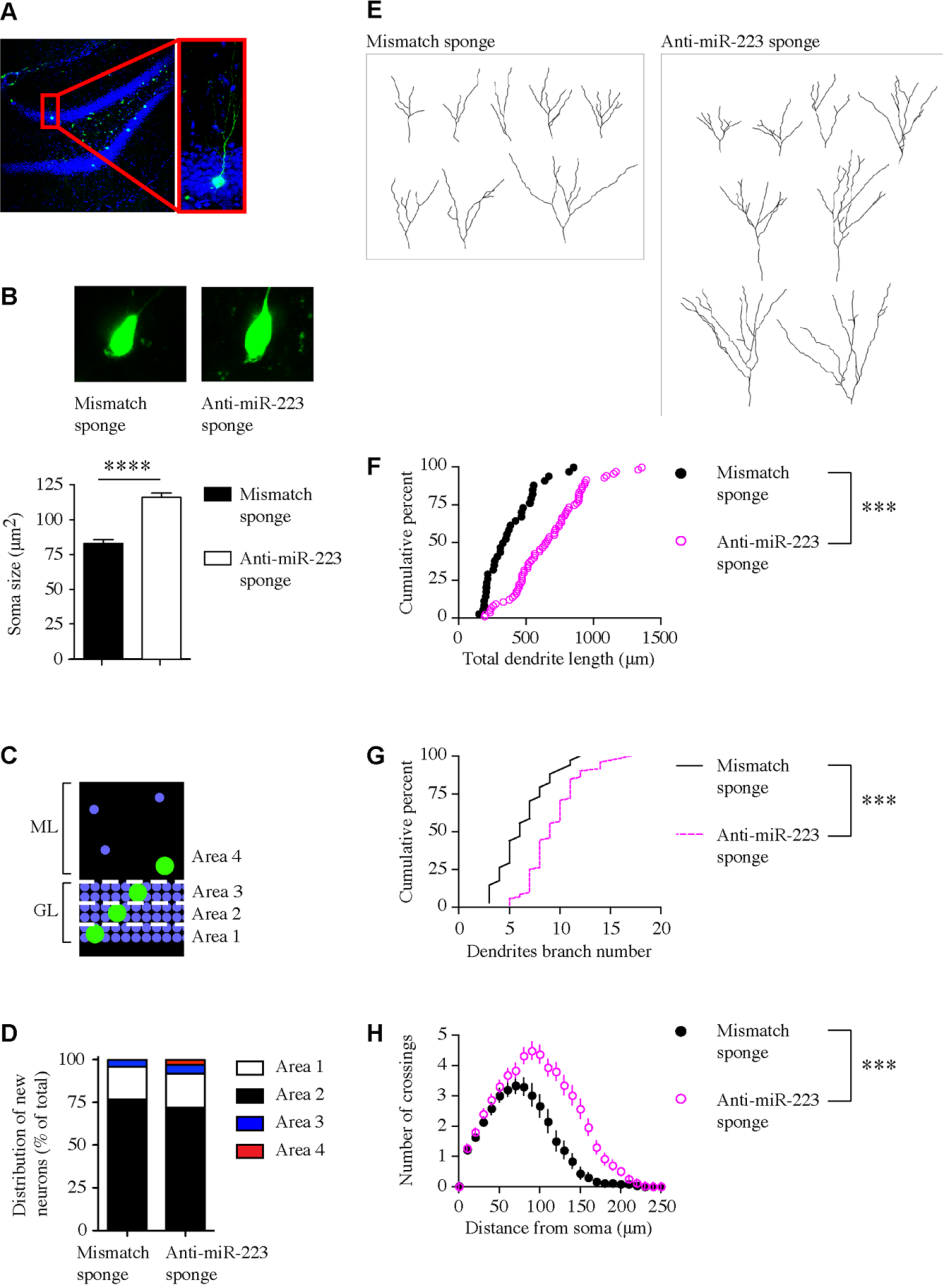
**Retrovirus-mediated single-cell soma size, migration and dendritic tree development analysis in dentate gyrus adult-born neurons. (A)** Confocal image of GFP + dentate gyrus granule cell. **(B)** Upper panel: Confocal image of GFP + dentate gyrus granule cell soma transduced with mismatch sponge or anti-miR-223 sponge retrovirus. Lower panel: Soma size quantification (****p < 0.0001, two-tailed *t*-test). **(C)** Diagram of dentate gyrus granule cell layer (GL) and molecular layer (ML) area determination. **(D)** Distribution of soma localization of GFP + adult born neurons in the dentate gyrus. **(B)**, **(D)** Mismatch sponge (*n* = 10) and anti-miR-223 sponge (*n* = 15). **(E)** Samples of 2D projection trajectories of the 3D confocal reconstructed images of the dendrites of GFP + neurons at 14 dpi (quantified in panels **F-H**). **(F)** Cumulative distribution plot of total dendrite length and **(G)** Cumulative distribution plot of dendrite branch number of neurons expressing sponge mismatch (*n* = 10) or anti-miR-223 sponge (*n* = 15). (***p < 0.001, Kolmogorov-Smirnov test) **(H)** Sholl analysis (dendritic complexity) of neurons expressing sponge mismatch (*n* = 10) or anti-miR-223 sponge (*n* = 15) (mean ± SEM). (***p < 0.001, Two way ANOVA). Data shown are representative of at least 3 independent experiments.

To investigate the cell-autonomous effect of miR-223 on immature neuronal morphogenesis, single-cell genetic analysis in the mouse adult dentate gyrus was performed. Retroviral vectors that target dividing NS/PCs in the adult dentate gyrus were engineered to express anti-miR-223 sponge or mismatch sponge with GFP reporter and injected into the adult mouse dentate gyrus. This approach allows for not only modulation of miR-223 function but also birth dating of adult neurons from NS/PCs in the dentate gyrus and permits lineage tracing using the GFP reporter. After 14 days post injection (14 dpi) GFP labeled granule cell neurons were detected in the dentate gyrus (Figure 2A). Adult born neurons expressing the anti-miR-223 sponge exhibited a significant increase in soma size compared to those expressing the control mismatch sponge (Figure 2B). Adult born neurons in the dentate gyrus mostly contribute to the basal granule cell layer (area 1, Figure 2C) and to a lesser extent to the mid-granule cell layer (area 2, Figure 2C). The anti-miR-223 sponge had no effect on distribution of soma localization in the dentate gyrus compared to the control mismatch sponge (Figure 2D). These results suggest that miR-223 regulates soma size development but not migration of the adult-born neurons in the hippocampus.

To explore the effect of miR-223 on adult-born neurons dendritic tree development, confocal z-stack imaging was used to reconstruct the 3-dimentional structure of the dendritic tree of single immature neurons using LSM-5 software. Anti-miR-223 sponge expression in the adult mouse NS/PCs led to a significant increase in immature neuron dendritic tree total length (Figure 2E,F), branch number per neuron (Figure 2E,G) and complexity as determined by Sholl analysis (Figure 2H) relative to neurons expressing the control mismatch sponge. These findings suggest that miR-223 regulates dendrite development in adult-born neurons in the hippocampus.

### MiR-223 regulates dendritic development in human neurons

**Figure 3:**
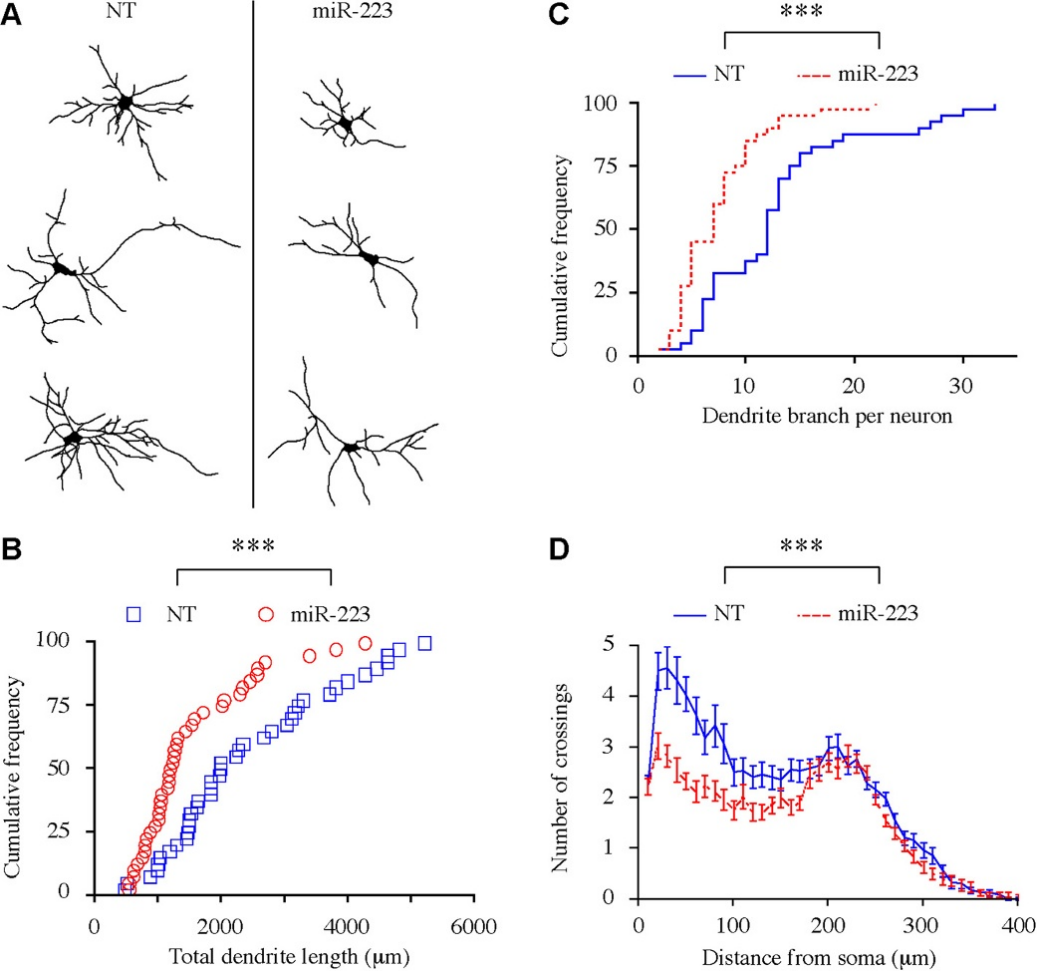
**Single-cell dendritic tree development analysis in ESCs-derived human neurons. (A)** Sample 2D projection trajectories of the dendrites of hESCs-derived Tuj-1-positive neurons. **(B)** Cumulative distribution plot of total dendrite length. (***p < 0.001, Kolmogorov-Smirnov test). **(C)** Cumulative distribution plot of dendrite branch number. (***p < 0.001, Kolmogorov-Smirnov test). **(D)** Sholl analysis of neurons dendrite complexity (***p < 0.001, Two way ANOVA). Data replicated 3 independent times. In each replicate; (NT) (*n* = 40), miR-223 (*n* = 40).

To test whether the effect of miR-223 on neuronal development in the adult mouse dentate gyrus occurs in human neurons, hESCs derived NS/PCs were differentiated into neurons as an in vitro model for human neurogenesis. NS/PCs were transfected with miR-223 expression vector or a non-targeting miRNA (NT) as a control. Following differentiation, the 3-dimensional structure of the dendritic tree of single Tuj-1-positive neurons was reconstructed. MiR-223 overexpression in human NS/PCs led to a significant decrease in developing neuron dendritic tree total length (Figure 3A,B), branch number per neuron (Figure 3A,C) and complexity (Figure 3D) compared to neurons expressing the NT control miRNA. On the other hand, Anti-miR-223 sponge overexpression led to a significant increase in immature neuron dendritic tree branch number per neuron and total dendritic arbor length (Additional file 2: Figure S1A, B). These findings suggest that miR-223 regulates dendritic development in human neurons.

### MiR-223 regulates NMDA induced calcium influx in human NS/PCs derived neurons

**Figure 4:**
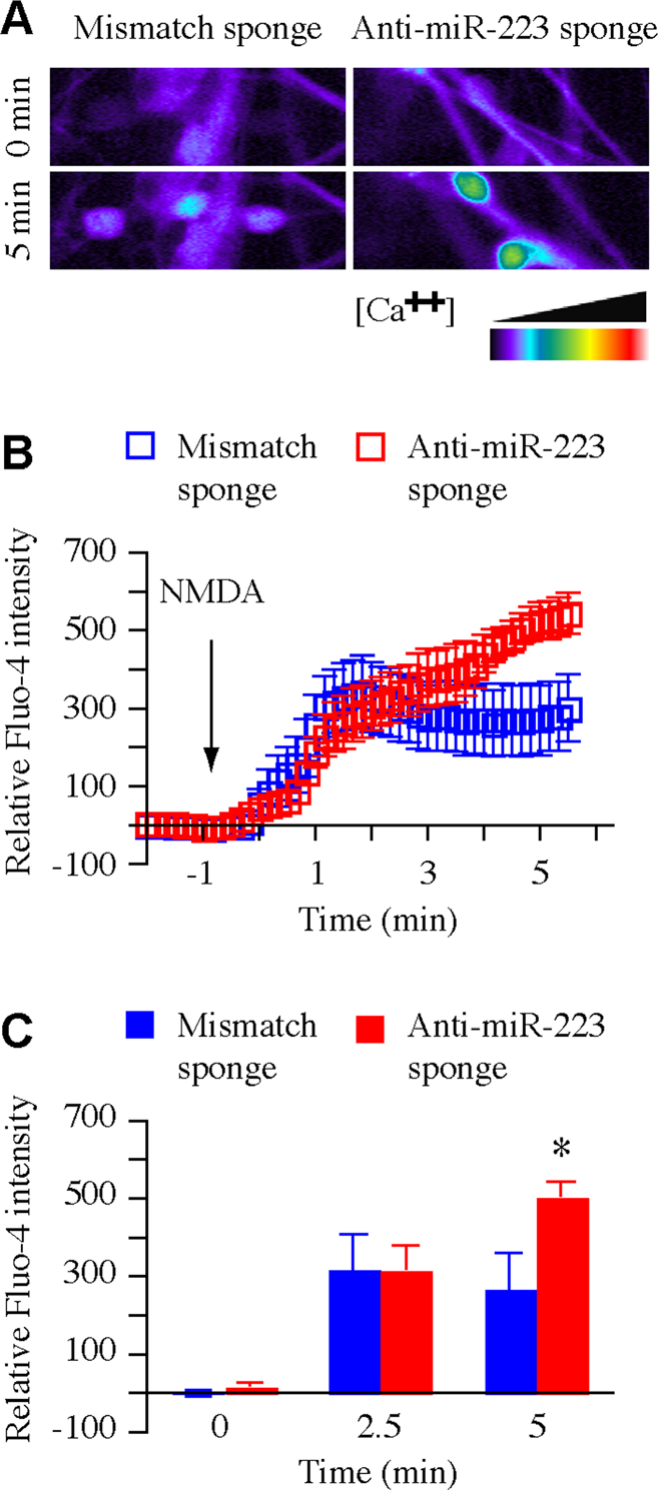
**Fluo-4 calcium mobilization assay in human NS/PCs derived neurons. (A-C)** Fluo-4 calcium mobilization assay (data represent 3 independent experiments) in hNS/PCs derived neurons transduced with lentiviral vectors expressing Anti-miR-223 sponge or mismatched sponge as a control. **(A)** Fluo-4 fluorescence intensity before (upper panels) and after (lower panels) 100 μM NMDA stimulation. **(B)** Relative intensity time course of fluo-4 fluorescence corresponding to calcium influx following NMDA stimulation. **(C)** Relative fluo-4 intensity quantification of indicated time points (*n* = 7, *p < 0.05, Student’s *t*-test).

MiR-223 regulates NMDA induced calcium influx in primary mouse hippocampal neurons [19]. Bioinformatics analysis suggested that miR-223 potentially regulates synaptic function in human neurons (Figure 1D). To evaluate the effect of miR-223 effect on human neuronal synaptic function Fluo-4 calcium mobilization assay for real-time calcium influx monitoring by live confocal imaging was used. Human NS/PCs derived neurons were transduced with lentiviral vector to express anti-miR-223-sponge or a mismatch sponge, which serves as a control. Inhibition of miR-223 by anti-miR-223-sponge leads to an enhanced calcium influx following NMDA stimulation compared to the mismatch sponge control group (Figure 4A-C). These findings suggest that miR-223 regulates NMDA-induced calcium influx in human neurons.

MicroRNAs are increasingly recognized as important players in regulation of neurogenesis [22]. Multiple microRNAs have been shown to regulate dendritic development such as: miR-132 [15, 23], miR-375 [13], and bantam [24]. While miR-223 is well studied as an immune system microRNA [25–30], others and we have demonstrated that miR-223 is expressed in the brain [19, 31, 32]. Furthermore, miR-223 was recently reported to inhibit neural cell specification [32]. The major finding of this study is the discovery of a new functional role for miR-223 in neuronal development. Inhibition of miR-223 enhances NMDA-induced calcium influx, leads to increased soma size, dendritic arbor length, branching and complexity but no change in migration in dentate gyrus immature neurons. Conversely, overexpression of miR-223 leads to decreased dendritic arbor length, branching and complexity in hESCs-derived neurons. Bioinformatics analysis suggested that miR-223 potentially regulates human neuron projection development, cell adhesion, synaptic function and the cytoskeleton (Figure 1D and Additional file 1: Dataset S1). Predicted targets of miR-223 in the mouse brain revealed similar results [19]. Post-transcriptional gene expression regulation is required for neuronal network formation including dendrite growth, branching and neuronal activity [33]. Regulation of NMDA-induced calcium influx by miR-223 in human neurons is consistent with our previous findings in primary mouse hippocampal neurons [19]. MiR-223 regulates the expression of the NMDA receptor subunit NR2B and the 2-amino-3-(3-hydroxy-5-methyl-isoxazol-4-yl)propanoic acid (AMPA) receptor subunit GluR2 [19]. Cumulative evidence in the literature links the NMDA receptor [34–37] and AMPA receptor activation [38–40] to dendrite growth and morphogenesis. In addition, NMDA receptor mediates integration of adult born neurons in the dentate gyrus neural circuit [41].

## Conclusions

These data suggest that miR-223 regulates soma size development, dendrite total length, branch number and complexity as well as neuronal activity. Taken together, these findings suggest that miR-223 regulates the integration of new-born neurons into neuronal circuitry.

While increasing the level of miR-223 might be beneficial as a neuroprotective strategy in the context of protection against neuronal cell death [19], this current study reveals a new context for potential translational applications exploiting miR-223. Inhibition of miR-223 is likely to be beneficial during neuroregeneration. Identifying small molecules inhibitors or antagomirs of miR-223 might carry a therapeutic potential in neuronal circuit repair conditions such as exogenous stem cell transplantation in the CNS or stimulation of endogenous neuroregeneration.

## Electronic supplementary material

Additional file 1: **Dataset S1.** DAVID functional annotation of brain-expressed sub-list of targetscan hsa-miR-223 predicted targets. TargetScan total predicted hsa-miR-223 targets, human target tissue expression distribution and functional annotation of brain-expressed human targets. (XLSX 248 KB)

Additional file 2: Figure S1: Single-cell dendritic tree development analysis in ESCs-derived human neurons. (A) Cumulative distribution plot of dendrite branch number. (***p < 0.001, Kolmogorov-Smirnov test). (B) Cumulative distribution plot of total dendrite length. (*p < 0.05, Kolmogorov-Smirnov test). Mismatch sponge (*n* = 84), anti-miR-223-sponge (*n* = 82). (TIFF 7 MB)
