# Supplementary figures and images for: MiR-223 regulates the differentiation of immature neurons
Source: Mol Cell Ther. 2014 Jun 17;2:18. doi: 10.1186/2052-8426-2-18 (PMC4229944; doi:10.1186/2052-8426-2-18)

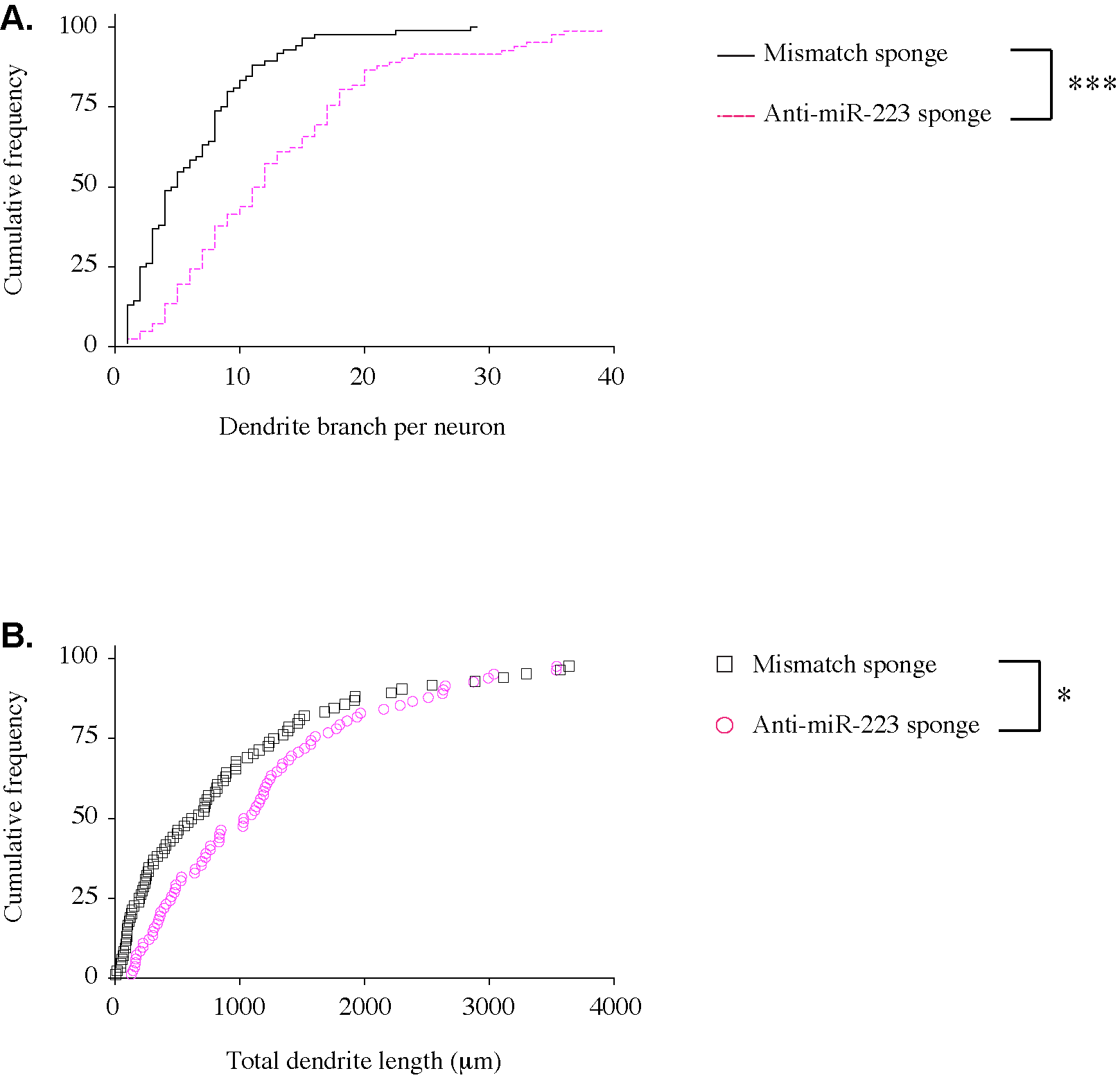

Supplement: Supplementary file 2 — Additional file 2: Figure S1: Single-cell dendritic tree development analysis in ESCs-derived human neurons. (A) Cumulative distribution plot of dendrite branch number. (***p < 0.001, Kolmogorov-Smirnov test). (B) Cumulative distribution plot of total dendrite length. (*p < 0.05, Kolmogorov-Smirnov test). Mismatch sponge (n = 84), anti-miR-223-sponge (n = 82). (TIFF 7 MB) [file 40591_2013_22_MOESM2_ESM.tiff]
